# Dynamic Changes in Liver Stiffness in Patients with Chronic Hepatitis B Undergoing Antiviral Therapy

**DOI:** 10.3390/diagnostics12112646

**Published:** 2022-10-31

**Authors:** Alin Lazar, Ioan Sporea, Alexandru Popa, Raluca Lupusoru, Diana Gherhardt, Ruxandra Mare, Alexandru Apostu, Madalina Hnatiuc, Roxana Șirli

**Affiliations:** 1 Division of Gastroenterology and Hepatology, Center for Advanced Research in Gastroenterology and Hepatology, “Victor Babeș” University of Medicine and Pharmacy, 300041 Timisoara, Romania; 2 Center for Modeling Biological Systems and Data Analysis, Department of Functional Sciences, Faculty of Medicine, “Victor Babeș” University of Medicine and Pharmacy, 300041 Timisoara, Romania; 3 Department of Cardiology, Division of Internal Outpatient Medicine, Prevention and Cardiovascular Recovery, Advanced Research Center of the Institute for Cardiovascular Diseases, “Victor Babeș” University of Medicine and Pharmacy, 300041 Timisoara, Romania

**Keywords:** hepatitis B virus, dynamic changes in liver stiffness, transient elastography, nucleos(t)ide analogues, oral antiviral therapy, FibroScan

## Abstract

This is a retrospective single-center study that included 87 subjects. All subjects had chronic hepatitis B or HBV cirrhosis and underwent nucleos(t)ide analogs (NUC) treatment for more than one year. The study aimed to evaluate the dynamic changes in liver stiffness (LS) measured by transient elastography (TE) during a median interval of 64 months. Patients were assessed prior to starting therapy and followed up annually. Liver stiffness measurements (LSM) were performed annually, and ten valid LSMs were obtained in each session. Reliable LSMs were defined as the median value of 10 measurements with Interquartile range/median (IQR/M) ≤ 30%. A significant decrease in liver stiffness values (*p* < 0.001) was observed during follow-up. In patients with liver cirrhosis, the LSMs decreased significantly after only one year, 24.6 ± 4.3 kPa vs. 13.5 ± 4.2 kPa (*p* = 0.007), whereas the decrease in non-cirrhotic patients was not significant, 7.31 ± 3.62 vs. 6.80 ± 2.41 (*p* = 0.27). Liver stiffness decrease was more significant in patients with initially higher transaminases. Undetectable viral load was achieved in 73.5% of patients in year one, 82.7% in year two, and 90.8% in year three of treatment. In conclusion, our study reveals a decrease in liver stiffness by TE in patients with chronic hepatitis B when undergoing anti-HBV therapy in the first two years. It can be used as a method for follow-up in patients undergoing NUC therapy.

## 1. Introduction

Infection with the hepatitis B virus (HBV) is the most common cause of chronic hepatitis. The World Health Organization (WHO) estimates that about 2 billion people have had contact with the virus. More than 400 million are hepatitis B surface antigen carriers, representing 5% of the world’s population [1]. HBV infection continues to be a problem worldwide, especially in developing countries, despite the existence of the HBV vaccine. Chronic hepatitis B can be associated with many clinical forms, from asymptomatic carriers to liver cirrhosis and hepatocellular carcinoma [2]. There is also a particular situation in the Asian region, where HBV infection is common, with a carrier rate of 10%; about a third of HBV patients will die due to chronic liver damage [3]. The goal of treatment in patients in the early stages of fibrosis is to prevent the development of liver cirrhosis. One of the first steps that should be performed in HBV patients is fibrosis severity assessment [4].

HBV treatment has advanced a lot over the past 15 years, but eradicating HBV remains a difficult target. Treatment with Pegylated interferon-alfa provides a response (serum HBV DNA levels under 2000 IU/mL for at least 12 months after the end of therapy) rate of 20–30%, and many patients are reluctant to undergo this treatment due to adverse effects and unfavorable profile. Therefore, oral antiviral therapy with nucleos(t)ide analogs (NUC) is the first line of treatment for most HBV patients [5].

Liver fibrosis must be evaluated in all patients with HBV since it predicts the risk of future liver-related morbidity and, consequently, the requirement for therapy, monitoring, and follow-up.

The actual techniques for diagnosing and staging liver fibrosis include invasive procedures such as liver biopsy followed by histological examination and non-invasive methods. Presently, ultrasound-based elastography techniques can be used to assess the physical properties of the liver tissue by studying tissue interactions with acoustically or mechanically generated energy (shear waves). Non-invasive ultrasound-based techniques for evaluating liver stiffness have become widely available. TE is one of the first and the most used and validated elastographic techniques for assessing liver stiffness as a marker of liver fibrosis in CLD. It was followed by the development of point share wave elastography (pSWE) and two-dimensional share wave elastography (2D-SWE) [6,7,8,9,10,11].

The EASL clinical practice guidelines recommend using TE in clinical practice, stating that TE can be considered the non-invasive standard for measuring liver stiffness [6,12]. TE is a fast, simple, reproducible non-invasive method of assessing fibrosis. There are some issues to consider when evaluating HBV patients by TE. Aminotransferase flares are recognized confounding factors for TE. In hepatitis B virus chronic infection, aminotransferase flares are frequent, leading to liver stiffness (LS) increase from 1.2 to 4.4 times as compared to baseline. Another factor is that HBV cirrhosis is macronodular, with less fibrous tissue than hepatitis C virus (HCV) cirrhosis; therefore, cut-off values for cirrhosis are lower in HBV infection than in HCV infection [13].

The study aimed to evaluate the dynamic changes of liver stiffness measured by TE in patients with chronic hepatitis B undergoing NUC therapy.

## 2. Materials and Methods

### 2.1. Study Population

This is a retrospective single-center study conducted from January 2011 to December 2018. We included 87 subjects with chronic hepatitis B or HBV cirrhosis, either naïve, non-responders, or relapses, to Peginterferon or Lamivudine therapy. All patients were initiated on NUC treatment and followed up for more than one year. During follow-up, liver stiffness measurements (LSM) were performed annually by using TE.

Treatment was initiated according to international guidelines [14]: in chronic hepatitis if the viral load was higher than 2000 UI/mL, and in patients with HBV cirrhosis, if HBV DNA was detectable, regardless of its value. Exclusion criteria were patients with less than 1-year follow-up, patients with chronic renal failure, patients younger than eighteen years old, patients with heavy alcohol consumption, patients with ascites, patients with biliary obstruction, patients with elevated aminotransferase levels more than five times the upper normal limit, patients with an oncological history or patients with focal liver lesions, patients with heart failure generating liver congestion, and patients with pacemakers. Blood samples were collected in the same session with LSM.

All patients signed informed consent and agreed to undergo liver stiffness measurements using TE and blood sampling. All measurements were performed by experienced operators (more than 100 LSM by TE).

Laboratory data such as alanine aminotransferase, aspartate aminotransferase, viral load, demographic data (age, gender), and previous treatment were noted. 

The study was conducted according to Helsinki declaration. The institutional ethics board approved the study design and protocol (NR. 12/05.12.2008). 

### 2.2. Transient Elastography (TE)

LS values were assessed using TE embedded in the FibroScan 502^®^ device (EchoSens, Paris, France). In each session, ten valid liver stiffness measurements (LSM) using either the M probe (standard probe with a transducer frequency of 3.5 MHz) or the XL probe (transducer frequency 2.5 MHz) were acquired. M and XL probes have been utilized according to the patient’s body mass index or depending on the skin-to-liver distance of each patient and according to the European recommendation on M and XL probe selection [9]. All patients were evaluated after fasting for at least 4 h. 

The measurements were performed in the right hepatic lobe by an intercostal approach, in an area free of large vessels or other structures, according to the EFSUMB and WFUMB guidelines, the patients being in dorsal decubitus with the right arm in maximum abduction [6,11,15]. After the measurement area was located, the operator pressed the probe button to start an acquisition. The software automatically rejects measurements that do not have the correct vibration form [16]. Reliable LSM was defined as a median value of 10 valid measurements with an interquartile range/median (IQR/M) ≤ 30% [16].

At the initial TE evaluation, the cut-off values used for discriminating among liver fibrosis stages were those proposed in the Young Eun Chon metanalysis [17]: 7.9 kPa for F ≥ 2, 8.8 kPa for F ≥ 3, and 11.7 kPa for F4. 

### 2.3. Statistical Analysis

Continuous data with a normal distribution were represented as the mean, standard deviation (SD); continuous data without a normal distribution were displayed as the median and interquartile range (IQR), and data for nominal variables were expressed as a percentage. The Kolmogorov–Smirnov test was used to test normality. The differences between groups were evaluated using the student *t*-test and analysis of variance (ANOVA) for continuous variables with normal distribution. To compare proportions, the Fisher test and Pearson chi-squared test were utilized. Microsoft Office 2019 and MedCalc Version 19.4 were utilized for the statistical analysis. At a confidence level of 95%, a *p*-value of 0.05 for intervals was considered significant.

## 3. Results

Baseline patients’ characteristics are presented in Table 1.

Regarding the response to treatment according to HBV-DNA serum levels, from the 87 patients with detectable HBV-DNA at baseline, undetectable HBV-DNA was achieved in 64 patients (73.5%) at year one, 72 (82.7%) at year two, 79 (90.8%) at year three. The patients were followed up for a median interval of 64 months (range 12–84).

**Figure 1:**
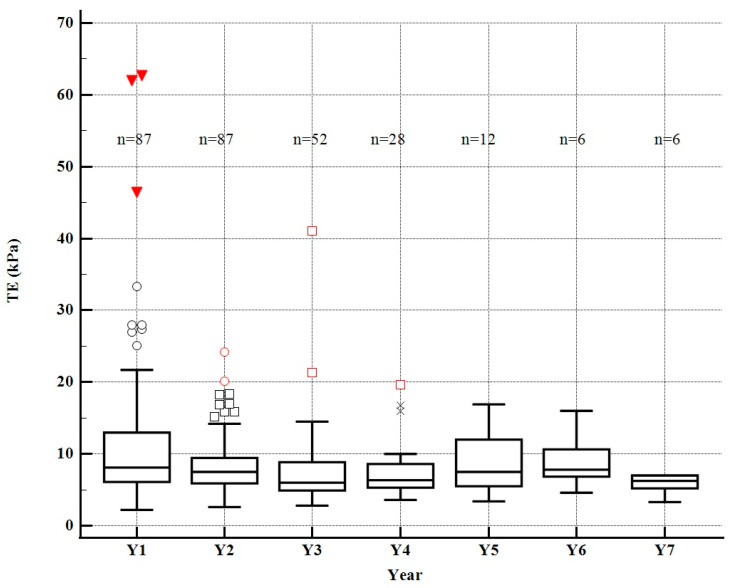
Dynamic of liver stiffness and patients during seven years. The red triangle, red/black circle, red/black square, black ex symbols represent outliers (an outlier is an extremely high or extremely low value that is located outside the whiskers of the box plot).

- 87 patients had at least two LSM made during the follow-up, and there was a significant decrease in the mean liver stiffness values from the first measurement vs. the second, 11.7 ± 8.4 kPa vs. 8.5 ± 4.1 kPa, *p* = 0.001 (Figure 1).
- 52 patients (59.7%) patients had at least three LSM, with a significant decrease in the mean values at the three assessments, 12.1 ± 11.0 kPa vs. 8.5 ± 4.3 kPa vs. 7.7 ± 3.9 kPa, *p* < 0.0001, but the post hoc tests showed that after the second year the decrease was not significant (*p* = 0.32).
- 28 patients (32.1%) had at least four LSM made during the follow-up, with a significant decrease in the mean values of the four LSM, 12.5 ± 8.3 kPa vs. 8.5 ± 4.9 kPa vs. 7.8 ± 4.3 kPa vs. 7.6 ± 4.6 kPa, *p* < 0.0001, but the post hoc tests showed that after the second year the decrease was not significant (second year LSM vs. third year LSM, *p* = 0.57; third year LSM vs. fourth year LSM, *p* = 0.86).
- 12 patients (13.7%) underwent at least five LSM throughout the follow-up, with significant variations between the five LSM, 14.8 ± 12.2 kPa vs. 9.1 ± 4.4 kPa vs. 8.4 ± 3.5 kPa vs. 8.7 ± 4.6 kPa vs. 8.0 ± 4.4 kPa, *p* < 0.0001. Starting with the second year, LSM remained relatively stable, which was demonstrated by the post hoc tests (second year LSM vs. third year LSM, *p* = 0.67; third year LSM vs. fourth year LSM, *p* = 0.85; fourth year LSM vs. fifth year LSM, *p* = 0.70).
- 6 patients (6.9%) had at least six LSM made during the follow-up, with significant differences between the six LSM, 17.0 ± 9.2 kPa vs. 9.5 ± 5.1 kPa vs. 8.5 ± 3.8 kPa vs. 9.3 ± 5.2 kPa vs. 8.8 ± 4.8 kPa vs. 8.6 ± 4.5 kPa, *p* = 0.01, but post hoc tests showed that after the second year the decrease was not significant (second-year LSM vs. third-year LSM, *p* = 0.70; third-year LSM vs. fourth-year LSM, *p* = 0.76; fourth-year LSM vs. fifth-year LSM, *p* = 86; fifth-year LSM vs. sixth-year LSM, *p* = 0.94).
- 6 patients (6.9%) had at least seven LSM made during the follow-up, with significant differences between the seven LSM, 7.6 ± 2.3 kPa vs. 6.4 ± 1.1 kPa vs. 6.1 ± 1.0 kPa vs. 6.0 ± 0.8 kPa vs. 5.7 ± 1.3 kPa vs. 5.7 ± 1.2 kPa vs. 5.0 ± 0.7 kPa, *p* = 0.04, but without any significant decrease after the second year, *p* = 0.56.

**Figure 2:**
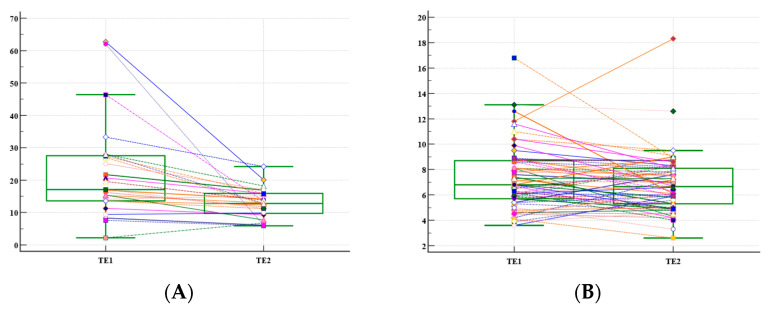
(**A**). Box plot of first two liver stiffness measurements (LSM) and dot and line diagram in liver cirrhosis patients, reflecting the liver stiffness decreases over one year; (**B**). Box plot of first two liver stiffness measurements (LSM) and dot and line diagram in non-cirrhosis patients, reflecting the liver stiffness decreases over one year; TE1 = Initial LSM by transient elastography (TE); TE2 = LSM by TE after one year. The different symbols and color lines represent the dynamic of the liver stiffness between the first two liver stiffness measurements of a patient.

When we divided the cohort into two sub-cohorts based on the initial evaluation of liver stiffness: cirrhosis (24 patients) vs. non-cirrhosis (63 patients), the LSMs significantly decreased in liver cirrhosis patients after only one year, 24.6 ± 4.3 kPa vs. 13.5 ± 4.2 kPa, *p* = 0.007. In contrast, in non-cirrhotic patients, the decrease was not significant after one year, 7.31 ± 3.62 vs. 6.80 ± 2.41, *p* = 0.27 (Figure 2).

Regarding the initial viral load, in patients with lower viremia—under 100.000 UI (38 patients), LSMs decreased significantly after one year of treatment: 13.2 ± 5.2 kPa vs. 8.6 ± 4.4 kPa, *p* < 0.0001; compared with the LSMs in patients with higher viremia—above 100.000 UI (49 patients): 9.7 ± 5.9 kPa vs. 8.4 ± 3.8 kPa, *p* = 0.25. 

When we divided the cohort according to the level of transaminases, in patients with values above the upper limit of normal (35 patients), LSMs decreased significantly after one year: 13.0 ± 5.8 kPa vs. 9.3 ± 4.3 kPa, *p* = 0.02; and in patients with normal transaminases (52 patients), LSMs also decreased, but with no statistical significance, 10.6 ± 9.5 kPa vs. 7.9 ± 3.8 kPa, *p* = 0.06. We also find a strong and direct correlation in the group with high transaminases at the beginning between the decrease in liver stiffness and the decrease of transaminases, r = 0.81, *p* < 0.0001.

## 4. Discussion

In the present study, patients with chronic hepatitis B undergoing NUC therapy were followed up using TE for a median period of 64 months. Subjects included had up to seven LSM measurements performed annually, during the follow-up period. A significant decrease in LSM values was observed during the follow-up period, the decrease in liver stiffness possibly due to the improvement of liver fibrosis. Liver stiffness is sensitive not only to fibrosis; it is also affected by various factors such as congestion, inflammation, steatosis, and cholestasis. Regardless of the cause, a decrease in liver stiffness due to initiating antiviral therapy has been associated with a favorable prognosis [18].

Several biomarkers can be used to assess antiviral efficacy, such as transaminases which can reflect the severity of liver inflammation and HBV DNA [19]. In our study, LSM by TE decreased significantly after one year of therapy in patients with higher levels of transaminases. The LSM decrease may also be influenced by liver inflammation decrease due to antiviral therapy. This explains why in our study, in patients with normal transaminases, where the inflammation was maybe not so severe, the liver stiffness decreased at one year, but without statistical significance. After one year of follow-up, there was a significant decrease in LSM in patients with lower initial levels of HBV DNA but not in patients with higher initial levels of HBV DNA; however, the patients with lower HBV DNA levels had higher initial liver stiffness values (13.2 ± 5.2 kPa) than those with high HBV DNA levels (9.7 ± 5.9 kPa). A further observation of our study was that the decrease in liver stiffness values was more significant in cirrhotic than in non-cirrhotic patients. 

A study evaluating long-term Entecavir therapy showed that 10 of 57 patients with Ishak score ≥4 had a median decrease of 1.5 points at 48 weeks compared to baseline. Four of the ten patients had an Ishak fibrosis score ≥5, and all of them demonstrated an improvement in the score with a median drop of 3 points. In the long-term rollover study, optional liver biopsy was offered after additional 48 weeks of treatment or, after a protocol amendment, for patients who had received at least three years of therapy. Most patients with CHB treated with Entecavir showed histological improvement and regression of fibrosis or cirrhosis [20].

Another study by Schiff et al. analyzed the course of histological fibrosis following long-term treatment with Entecavir (approximately six years) in 10 Nucleoside-naive patients with a mean Ishak score of 4.6. An improvement of at least one point was found in all patients evaluated after long-term therapy. The mean change from baseline was −0.8 points after 48 weeks of treatment, compared to −2.2 points after long-term therapy [21].

In a study by Marcellin et al., which examined a cohort of 348 patients with HBV infection treated with tenofovir disoproxil fumarate over 5 years, 38% of patients had an Ishak score greater than 4. Still, this proportion decreased to 28% after the first year of treatment and to 12% in the 5th year of treatment. Altogether, regression of fibrosis was observed in 51% of patients and histological improvement in 87% of patients after five years. A number of 96 patients had cirrhosis, with an Ishak score ≥5 at the beginning of the treatment; 71 out of these 96 patients had a reduction in fibrosis after five years of treatment, and more than half (56 patients) had a reduction of at least three units of the score [22]. This study concluded that NUCs were effective in long-term treatment and could lead to fibrosis regression, significantly improving the prognosis in patients with cirrhosis [23].

In a study published by Won Jang et al., a significant decrease in LSM values was observed at a median assessment interval of 16 months. The main factor associated with liver stiffness decrease was achieving a complete virologic response [24]. Furthermore, a higher initial LSM value contributed to a more significant LSM reduction in chronic hepatitis B patients after Entecavir therapy [25].

Another two studies showed that liver stiffness, measured by TE, significantly decreased after the initiation of NUCs. The first study, which included a group of 44 patients with chronic hepatitis B undergoing antiviral treatment, showed a significant decrease in LSM values in the first three years after the initiation of therapy, followed by a steady transition from 3 to 5 years [26]. In the second study, Enomoto et al., which included 50 patients, LSM values decreased from 11.2 kPa to 7.8 kPa during 12 months of treatment with Entecavir [27]. Similar results were observed in our study, where liver stiffness values decreased during NUCs treatment, with the most significant decrease in the first two years.

In another study, this time on HCV patients, liver stiffness values also decreased in HCV liver cirrhosis patients treated with direct-acting antiviral (DAA) agents. At the end of treatment (EOT), the mean liver stiffness values of the study group considerably decreased as compared to baseline: 26.4 ± 11.7 vs. 23.5 ± 13.3 kPa (*p* = 0.01) [28]. 

A systematic review and meta-analysis in patients with hepatitis C demonstrated that in those with a sustained virologic response (SVR), LSM by TE decreased by 2.4 kPa at the end of therapy, by 3.1 kPa at 1–6 months from EOT, by 3.2 kPa at 6–12 months and by 4.1 kPa after 12 months of treatment. The patients with liver cirrhosis had the most significant decrease [29]. In our study, liver stiffness decrease was the most remarkable in cirrhotic patients after one year of treatment.

By reflecting the variations in liver necroinflammation, TE is a valuable technique for monitoring the response to antiviral therapy [30,31]. In a study by Zeng et al., which included 175 patients with chronic hepatitis B receiving antiviral treatment, 67 were evaluated by TE and liver biopsy at the start of treatment and 96 weeks of treatment. The results showed an improvement in the METAVIR score at 96 weeks compared to the baseline score (*p* < 0.001), with 50 patients (74.6%) having a significant histological response. After 96 weeks of treatment, the median decrease in stiffness values was 3.6 ± 2.9 kPa (2.4–11.8 kPa). The AUROC of TE decrease for predicting improved liver fibrosis was 0.68 (*p* = 0.029). A drop of liver stiffness values by at least 4.1 kPa had 88.2% sensitivity and 50% specificity for predicting an improvement in liver fibrosis severity [31].

Lim et al. performed a study that included 62 naive HBV patients treated with Entecavir for at least 12 months. TE was performed at the start of treatment and after 48 weeks of treatment, and a liver biopsy was performed in 15 patients at baseline and during treatment after a median time interval of 25.5 (13.1–36.6) months. The median liver stiffness values significantly decreased at 48 weeks of treatment: 8.8 kPa (3–33.8 kPa) as compared to baseline, 15.1 kPa (5.6–75 kPa) (*p* = 0.012). The results of this study also show a mean decrease of 6.4 ± 3.2 points in the necroinflammation scores compared to baseline and a decrease of 0.4 ± 0.6 points in terms of fibrosis scores. Furthermore, there was a strong correlation between the decreased ratio of liver stiffness and the decreased ratio of necroinflammatory and fibrosis scores (r = 0.668, *p* = 0.007) [30].

All the studies mentioned above have shown that successful antiviral therapy, both in HBV and HCV patients, leads to an improvement in liver histology regarding both inflammation and fibrosis, which is reflected in an improvement in liver stiffness measurements by means of TE. Our study also revealed a significant decrease in liver stiffness values in patients with chronic hepatitis B undergoing anti-HBV treatment. 

Our study has some limitations. We evaluated patients for liver fibrosis only non-invasively using TE and not by using liver biopsy. However, in clinical practice, in large cohorts, it is challenging to perform serial biopsies (before and after treatment) for follow-up for a long period. Another limitation is that we did not use another non-invasive method to quantify inflammation, and its decrease was possibly another cause of the continuous decrease in liver stiffness. Another significant issue is the absence of data on liver function tests throughout follow-up.

The most important finding of this study was that liver stiffness decreased in the first 2 years following the initiation of antiviral therapy, as liver stiffness is affected by both hepatic fibrosis and the necroinflammation underlying viral replication. Following cessation of use, alcoholic patients exhibited comparable changes in stiffness. During the follow-up period, it is essential to evaluate hepatic stiffness in order to monitor disease progression and therapy efficacy. 

However, consistent with the studies discussed above, our study showed a decrease in hepatic stiffness due to antiviral therapy.

## 5. Conclusions

In conclusion, our study reveals that liver stiffness assessed by TE decreased significantly in the first two years in patients with chronic hepatitis B when undergoing anti-HBV treatment.

## Figures and Tables

**Table 1:** Patient’s characteristics.

Parameter	N (%), Median (Range), Mean ± SD
Age (years)	49.9 ± 13.1
Gender	
Men	67 (70.1%)
Women	20 (22.9%)
ALT (IU/mL)	29 (10–210)
AST (IU/mL)	26 (7–225)
HbeAg positive	12 (13.7%)
Naïve	47 (54.0%)
Fibrosis stage at inclusion based on LSM by TE	
F0–1	42 (48.2%)
F2	11 (12.6%)
F3	10 (11.5%)
F4	24 (27.7%)

N = total number; SD = standard deviation; ALT = alanine aminotransferase; AST = aspartate aminotransferase; LSM = liver stiffness measurements; TE = transient elastography.

## Data Availability

Not applicable.
